# A randomized controlled trial of group CBT with positive psychotherapy intervention for university students with maladaptive perfectionism in China

**DOI:** 10.3389/fpsyg.2023.1161575

**Published:** 2023-07-19

**Authors:** Zhijuan Zuo, Xingli Zhang

**Affiliations:** ^1^Mental Health Center in Department of Student Affairs, Yunnan University, Kunming, China; ^2^Key Laboratory of Behavioral Science, Institute of Psychology, Chinese Academy of Sciences (CAS), Beijing, China; ^3^Department of Psychology, University of Chinese Academy of Sciences, Beijing, China

**Keywords:** group CBT with positive psychotherapy intervention, maladaptive perfectionism, randomized controlled trial, university students, China

## Abstract

**Objective:**

The primary objective of this randomized controlled trial was to find a more economical and feasible intervention for maladaptive perfectionism, which is a risk and maintenance mechanism for various psychopathologies.

**Methods:**

In total, 64 university students who met the total inclusion criteria were randomly assigned to either group CBT with positive psychotherapy intervention or a 16-week waitlist (WL). The intervention group received 2 h of therapy for eight weekly sessions. Measurements of maladaptive perfectionism and the symptoms of depression and anxiety were conducted at baseline, post-intervention, and follow-up.

**Results:**

There was no statistically significant difference in the scores of the Frost Multidimensional Perfectionism Scale, Self-Rating Depression Scale, and Self-Rating Anxiety Scale between the intervention group and the waitlist group at baseline (all *p* > 0.05). The intervention group had a significant main effect of time and a time × group interaction effect of the maladaptive aspects of perfectionism, Concern over Mistakes and Doubts about Actions, and depression and anxiety scores in comparison with the waitlist group at the post-intervention and 8-week follow-up and had no statistically significant effects on the scores for Personal Standards, Parental Expectation, and Parental Criticism. The analysis showed that group intervention reduced symptoms of depression and anxiety while reducing maladaptive aspects of perfectionism.

**Conclusion:**

This study added to the literature on CBT interventions for maladaptive perfectionism and indicated that group CBT with positive psychotherapy intervention had substantial long-term effects on the maladaptive perfectionism of university students in China. Moreover, the results indicated that the intervention increased participants’ self-acceptance.

## Introduction

Perfectionists can be described as those whose standards are high beyond reach or reason, people who strain compulsively and unremittingly toward impossible goals and who measure their own worth entirely in terms of productivity and accomplishment according to Burns (Lo and Abbott, 2013). The multidimensional construct of perfectionism has been well-established by psychology researchers. Through percentile, median, or clustering methodology, scales of perfectionism divide people into three categories: adaptive perfectionism, maladaptive perfectionism, and nonperfectionism (Yang et al., 2010). Maladaptive perfectionists are conceptualized as experiencing greater levels of distress as they strive to meet unrealistic standards and unattainable goals. They are driven by a fear of failure and criticism and attempt to escape feelings of inferiority (Lo and Abbott, 2013).

Researchers have continued to demonstrate clear associations between perfectionism and personal adjustment problems, including depression disorders, anxiety disorders, and eating disorders, during the past two decades (Lo and Abbott, 2013). Perfectionism is not only elevated across many disorders when compared to healthy controls but is also a transdiagnostic process and is implicated as a risk and maintenance mechanism of various psychopathologies (Galloway et al., 2022). Perfectionism is a complex personality style that encompasses all aspects of one’s behavior and is associated with myriad and marked psychological, physical health, relationship, and achievement problems (Hewitt et al., 2017).

Maladaptive perfectionism may positively predict the symptoms of psychiatric disorders and impede the success of treatment for these disorders (Rhéaume et al., 2000). For example, the adverse outcome of standard treatment for depression could be predicted by the level of maladaptive perfectionism before treatment, the reduction in depressive symptoms at the end of treatment and follow-up was negatively correlated with perfectionism, the treatment effect on social anxiety was reduced by perfectionism, and the ability of OCD patients to finish exposure, blocking response, and cognitive reconstruction tasks was interfered with by perfectionism (Steele et al., 2013). Despite the important role maladaptive perfectionism plays in psychopathology and treatment, few interventions have targeted it (Ong et al., 2019). According to some researchers’ views, ignoring perfectionism can be a barrier to psychotherapy and undermine effectiveness (Mitchell et al., 2013).

Targeting perfectionism in treatment resulted in better psychological outcomes than targeting each maintenance factor of the psychiatric disorder (Lo and Abbott, 2013). A recent meta-analysis of eight studies found that it was possible to significantly reduce levels of maladaptive perfectionism using short cognitive behavioral interventions (Lloyd et al., 2015). Targeting maladaptive perfectionism may be effective in reducing symptoms across a range of disorders (Lloyd et al., 2015).

Individual CBT (ICBT) was mostly used in past studies, while group interventions were rare. The interventions published in international journals were mostly conducted in English-speaking samples, and only two interventions were conducted in Hispanic samples (Bento et al., 2017; Matos and Steindl, 2020). There is still a lack of a detailed theoretical and psychological understanding of the psychopathological processes in perfectionism associated with psychosis to explain how the changes occurred in treatment for the different cultural backgrounds (Gaudiano, 2005).

The relationship between maladaptive perfectionism and distress has been analyzed by many researchers in China, and interventions have been designed mainly for nonclinical samples. In addition to cognitive behavioral therapy (Zheng, 2014), other therapies included mindfulness (Zhu, 2014), solution-focused brief therapy (Xu, 2019), sandplay therapy (Yu, 2012), and other therapies (Liu, 2015; Zeng, 2018; Wang, 2019). However, these interventions were not normative because they were mostly implemented by graduate students for their dissertation research. The inclusion criteria of participants were not set, and clinical performance of maladaptive perfectionism or related problems such as rumination (Zhu, 2014; Zeng, 2018), procrastination (Zheng, 2014), examination anxiety (Liu, 2015), self-efficacy (Xu, 2019), mental resilience (Wang, 2019), and psychological security perception (Wang, 2019) were mainly aimed at in most interventions. Group tutoring activities were more common, and therapeutic interventions were less common. Effect size data for maladaptive perfectionism, depression, and anxiety were absent in all these interventions. The number of participants was not enough to meet the requirements of a larger effect size (Zheng, 2014; Zhu, 2014; Zeng, 2018). Intervention effects upon follow-up were tracked in only one study (Zeng, 2018). Therefore, it was difficult to prove that the symptom reduction in psychiatric disorders was related to the change in maladaptive perfectionism and how the intervention effect was maintained during the follow-up.

Students may be an at-risk population, as shown by fruitful academic research (Arana et al., 2017). Nearly two-thirds of students can be categorized as perfectionists, with over a quarter considered maladaptive perfectionists (Grzegorek et al., 2004).

The notion of perfectionism is believed to be particularly relevant to the Chinese population (Wang et al., 2007). Despite the fact that interdependence is a cultural value, it still causes increased liability for the maladaptive aspects of perfectionism and augmented the association of perfectionism with depressive symptoms (Dibartolo and Rendón, 2012). Empirical research has found that Asian Americans report high levels of both parental expectations and criticism relative to other ethnic groups. The same tendency exists in Chinese parents (Yin et al., 2022) as Chinese children are nurtured to be very sensitive to mistakes and failures (Fong and Yuen, 2011), and scores in maladaptive perfectionism of university students are higher and more strongly associated with depression in Asian cultures. In the practice of psychological counseling, researchers have found that many university student clients who had not met the diagnostic criteria of a psychiatric disorder had a high perfectionism tendency due to their parents’ high expectations or frequent criticism. Positive changes occurred relatively slowly in these individuals with counseling. It is necessary to find a way to reduce the levels of maladaptive perfectionism in Chinese university students.

Unrealistically high standards and excessive focus on mistakes are cognitive biases of perfectionists, and it is not easy to change this cognitive habit consciously. Perfectionism is typically ego-syntonic, and individuals may show resistance to changing what they view as a part of their personality (Ong et al., 2019). The serious resistance problems in the treatment of extreme perfectionists were as follows in this study: fear of failure, rigid adherence to their inflexible standards, and self-punishment (Zhang et al., 2010). Positive psychotherapy can increase the pleasant experience of participants and replace the habitual negative experience to enhance the motivation for change in perfectionists. However, there are few available studies in which positive psychotherapy was used to target perfectionism treatment.

Therefore, the primary aim of this RCT was to examine whether group CBT with positive psychotherapy intervention was superior to the waitlist condition in reducing maladaptive perfectionism, depression, and anxiety and whether the intervention effects can be maintained during follow-up.

## Method

### Participants

G*Power 3.1.9.2 was used for sample size calculation, and the effect size criteria were proposed by Cohen (0.8 for large effect, 0.5 for medium effect, and 0.2 for small effect) (Zhao and Wang, 2019). In this study, the effect size was Cohen’s *d* = 0.8, one-sided alpha = 0.05, power = 0.90, and a minimal sample size of 56 participants (28 in each group) would be needed.

Volunteers were recruited through advertisements on the public course of Mental Health Education for university students and public accounts of mental health centers on WeChat. The inclusion criteria of participants were as follows: (a) being an undergraduate or postgraduate student; (b) willingness to participate in group intervention; (c) 84 or above total score in the FMPS (an individual with a score above 84 was defined as high perfectionism tendency by Frost); (d) 53 ≤ standard score in the SDS ≤ 72, or 50 ≤ standard score in the SAS ≤ 70; and (e) commitment to participate in group intervention to the end. Exclusion criteria were (a) hallucinations or other acute episodes of psychosis; (b) immediate risk of suicide, self-injury or injury to others; (c) any current psychological treatment for psychiatric problems; and (d) any change in psychotropic medication 3 months prior to entering treatment.

Participants were informed that they should not attend any other form of therapy and if there was a need to do so, they should inform the investigators.

### Procedures

Individuals interested in participating scanned the QR code for enrolment. This resulted in 96 students enrolling and following the enrolment measurements with the FMPS, SDS, and SAS. Of these, 80 met the score requirements and 72 took part in a face-to-face interview of 15 min according to appointment. The enrolment criteria were checked during the interview. Three students dropped out because of the change in course time, and five students did not meet the total enrolment criteria. Then, 36 female participants who met all the enrolment criteria were randomized using a random numbers generator in a 1:1 ratio to one of two conditions: intervention (n = 18) or waitlist (n = 18). Similarly, 28 male participants who met all the enrolment criteria were randomized using a random numbers generator in a 1:1 ratio to one of two conditions: intervention (*n* = 14) or waitlist (*n* = 14).

**Figure 1:**
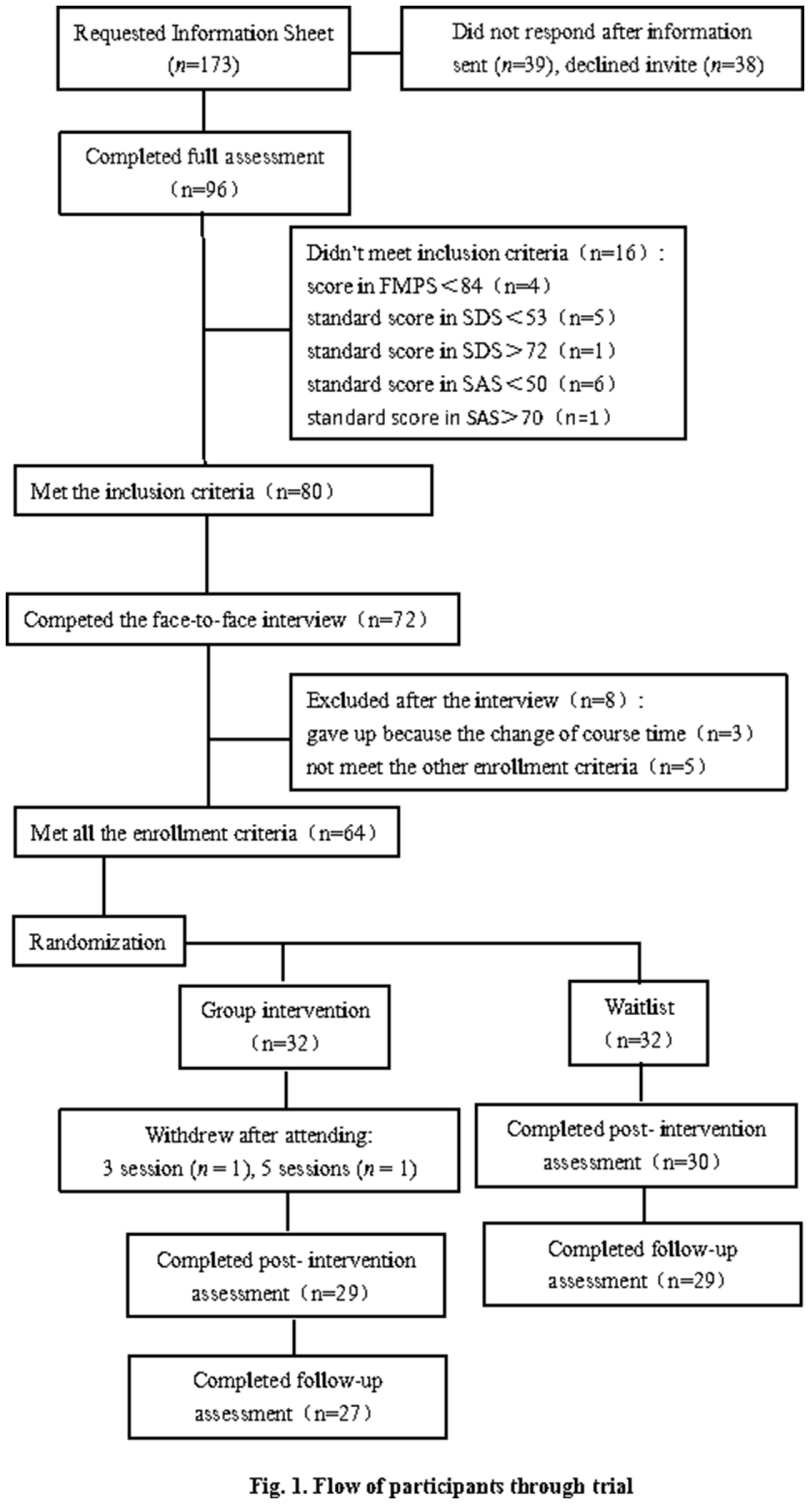
Flow of participants through the trial.

Because there is no Ethics Committee in the university where the research was conducted, the participants were formally invited to take part in the intervention, and formal informed consent documents were given to participants to sign. Participants could choose to quit if they felt that the study was not good for their recovery. They were also informed about the purpose of the study, the risks and benefits of the group intervention, the design of the intervention, and confidentiality principles. The flow of participants through the trial is shown in Figure 1.

Measurement scales were distributed to participants at baseline (T1), post-intervention (immediately after intervention)(T2), and at an 8-week follow-up (T3). Scales were distributed to the intervention group face to face by irrelevant assistants in the Mental Health Center of Yunnan University, while the waitlist group had made an agreement in advance to complete scales on an online database and was reminded by assistants with messages at a fixed time. Data was also collected by these assistants. All data was used for research purposes only. Paper scales and informed consent documents were kept in the archives of the Psychological Center at Yunnan University. Personal information had been deleted from the online original data.

### Measures

#### Frost multidimensional perfectionism scale

Perfectionism was measured by the Frost Multidimensional Perfectionism Scale (FMPS). It has been widely used, and demonstrated satisfactory reliability and validity (Limburg et al., 2017). It is a 35-item measure consisting of Concern over Mistakes (CM), Personal Standards (PS), Doubts about Actions (DA), Parental Expectation (PC), Parental Criticism (PE), and Organization (OR) (Frost et al., 1990). Four FMPS subscales (CM, DA, PC, and PE) measure maladaptive aspects of perfectionism (MFMPS) (Slade et al., 2009). In total, 35 items of FMPS are rated on a five-point Likert scale ranging from 1 (strongly disagree) to 5 (strongly agree). The internal consistencies of subscales range from 0.77 to 0.93 (Egan and Hine, 2008).

An FMPS translation translated by Cheng was used in this study, and the translation was slightly modified based on Chinese language habits by Zifei. The Cronbach’s alpha values of the subscales range from 0.64–0.83 (Cheng et al., 1999; ZI and ZHOU, 2006). It had good internal consistency (α of subscales range from 0.74–0.91) in this sample.

#### Self-rating depression scale

The severity of depression was measured by the Self-Rating Depression Scale (SDS). SDS was developed by Zung in 1965. This self-report scale consists of 20 items rated on a four-point Likert scale (no or very little of the time, some of the time, a good part of the time, and most of the time or all the time). Participants rated how much they have experienced symptoms of depression over the past week. Its Cronbach’s alpha value was 0.82. According to the Chinese norm, a score below 53 indicates no depression, 53–62 indicates mild depression, 63–72 indicates moderate depression, and 73 or above indicates severe depression (Thi Thuy Ta, 2019). It has good validity and acceptable internal consistency in this study (α = 0.70).

#### Self-rating anxiety scale

The severity of anxiety was measured by Self-Rating Anxiety Scale (SAS). SAS was developed by Zung in 1971. It is a 20-item, 4-point self-rating scale designed to assess the subjective feelings of anxious patients. Studies proved that SAS has a good evaluation effect on adults with anxiety symptoms, and Cronbach’s alpha was 0.81. According to the Chinese norm, a score below 50 indicates no anxiety, 50–59 indicates mild anxiety, 60–70 indicates moderate anxiety, and 71 or above indicates severe anxiety (Guo, 2019). The SAS has good reliability in the current study with α = 0.72.

### Intervention

The intervention was conducted by a licensed counselor of the Clinical and Counselling Psychology Registration System (CCPRS) in China and assisted by 3 graduate students majoring in psychology or social work. The counselor had practiced individual psychological counseling and group intervention for more than 15 years. The group intervention consisted of eight weekly 120-min sessions.

Based on the scientific intervention design of past literature, the structure of the intervention was adapted from the books “Never Good Enough” (Basco, 2000), “The Principles of Cognitive Behavioural” (Beck, 2013), and “Positive Psychotherapy” (Yang, 2010), and from research articles entitled “A comparative study of group positive psychotherapy and group CBT in the treatment of depressive disorders” (Furchtlehner et al., 2020), and “Cognitive-behavioral group therapy in major depressive disorder with focus on self-esteem and optimism” (Moloud et al., 2022). Resistance problems such as “fear of failure,” “rigid personal high standards,” and “doubt of action” were targeted in the intervention, and the tendency of self-punishment was decreased by changing the participants’ self-schema.

In the first part, the problem behaviors related to perfectionism were checked, and a list was made. The second part consisted of recognizing the negative automatic thinking of perfectionism, learning to understand the influence of automatic thinking on their emotions and behaviors (the A-B-C theory), and accepting that their beliefs can be changed. The third part consisted of behavioral experiments and cognitive reconstruction to challenge cognitive distortions and how to change them. The fourth part focused on procrastination, which is a prominent problem behavior in perfectionism, and introduced four ways to change procrastination habits, and developed and implemented plans for immediate action. Positive psychotherapy was added in the following intervention. The fifth part consisted of recognizing the meanings of mistakes and failures from a positive perspective and reconstructing the past mistakes and failures of participants. The sixth part consisted of deliberate practices on learned optimism, including extending the definition of success, hope thinking, and process orientation. During the seventh part, the source of perfectionism (the influence of inheritance and family education) was explored, the barriers to self-esteem caused by cognitive distortions were recognized, and core beliefs were reconstructed (find the strengths that perfectionism can bring to participants). In the eighth part, the strength development plan was developed, and role-play techniques to exercise self-confidence and determination were practiced. In the end, the participants learned to view themselves with a new self-schema.

### Data and statistical analyses

Data were collected from participants who were willing to complete baseline, post-treatment, and follow-up assessments. To compare the main effects and interaction effects between the intervention and waitlist groups ANCOVA analyses were conducted using the SPSS 20.0.

Paired t-tests were used to ascertain changes in the measures between baseline, post-intervention, and 8-week follow-up. Independent t-tests were used to assess differences between intervention and waitlist participants at baseline, post-intervention, and 8-week follow-up. Cohen’s d statistic served as effect size by using JASP.

## Results

### Sample descriptive

The participation rate was high in the intervention group, and 30 students (94%) took part in all eight sessions. In total, 27 students (84%) completed all the scales at baseline, immediately following the intervention, and at the 8-week follow-up, including 11 males and 16 females. In the waitlist group, 29 students (91%) completed all the scales, including 11 males and 18 females. The demographic characteristics of the participants are shown in Table 1.

**Table 1:** Sociodemographic characteristics of participants at the pre-treatment assessment.

	Overall (*N* = 56)	intervention group (*n* = 27)	Waitlist (*n* = 29)
Age	M = 21.1 (SD = 2.2)	M = 21.4 (SD = 2.3)	M = 20 (SD =2.1)
*Gender*			
Males	22 (39.3%)	11 (40.7%)	11 (37.9%)
Females	34 (60.7%)	16 (59.3%)	18 (62.1%)
*Highest educational level*			
Bachelor’s degree	35 (62.5%)	16 (59.3%)	19 (65.5%)
Master’s degree	21 (37.5%)	11 (40.7%)	10 (34.5%)
*Marital status*			
Single	45 (80.4%)	22 (81.5%)	23 (79.3%)
Married/partner	11 (19.6%)	5 (18.5%)	6 (20.7%)
Previous psychological treatment	8 (14.3%)	3 (11.1%)	5 (17.2%)
Previous psychotropic medication	5 (8.19%)	2 (7.4%)	3 (10.3%)

### Data and statistical analyses

Means and standard deviations (SD) of dependent variables at baseline, post-intervention, and 8-week follow-up are presented in Table 2. Baseline assessment indicated that all the participants experienced moderate depression (mean = 60.50, *SD* = 4.80) and moderate severe anxiety (mean = 62.34, *SD* = 5.80) on the SDS and SAS, respectively. Also, the mean score on the FMPS (mean = 109.91, *SD* = 13.41) suggested major difficulties in perfectionism.

**Table 2:** Estimated means, standard deviations, main effect of time, and time*group interaction effect for each outcome measure divided by condition and assessment.

	Group	Baseline	Post-intervention	Follow-up	Main effect of time *F*-value (*p*, η2)	Time*group interaction *F*-value (*p*, η2)
CM^a^	CBT-P^i^	21.74 (7.55)	17.00 (4.88)	17.81 (5.97)	15.03 (*p* < 0.001) (biasη2 = 0.36)	16.28 (*p* < 0.001) (biasη2 = 0.38)
	Waitlist	21.10 (5.19)	21.76 (4.99)	21.21 (4.88)		
PS^b^	CBT-P	24.07 (4.85)	23.78 (4.20)	23.56 (4.52)	1.91 (*p* = 0.159) (biasη2 = 0.07)	1.74 (*p* = 0.186) (biasη2 = 0.02)
	Waitlist	23.62 (3.52)	23.03 (4.36)	23.48 (3.72)		
PE^c^	CBT-P	15.52 (4.00)	15.07 (4.13)	15.11 (4.10)	1.04 (*p* = 0.362) (biasη2 = 0.04)	1.74 (*p* = 0.185) (biasη2 = 0.06)
	Waitlist	16.28 (4.36)	16.00 (3.71)	16.34 (3.79)		
PC^d^	CBT-P	9.52 (2.91)	9.19 (3.10)	9.37 (2.86)	0.62 (*p* = 0.542) (biasη2 = 0.02)	1.59 (*p* = 0.214) (biasη2 = 0.06)
	Waitlist	9.24 (2.29)	9.62 (1.72)	9.62 (1.59)		
DA^e^	CBT-P	13.81 (3.76)	11.33 (2.90)	12.00 (2.87)	3.91 (*p* = 0.026) (biasη2 = 0.13)	5.75 (*p* = 0.005) (biasη2 = 0.18)
	Waitlist	13.28 (2.53)	13.52 (2.89)	13.55 (2.43)		
MFMPS^f^	CBT-P	60.59 (12.70)	52.59 (9.68)	54.30 (10.32)	14.11 (*p* < 0.001) (biasη2 = 0.35)	23.78 (*p* < 0.001) (biasη2 = 0.47)
	Waitlist	59.90 (8.90)	60.90 (8.84)	60.72 (8.88)		
SDS^g^	CBT-P	60.03 (6.23)	55.51 (6.34)	56.57 (5.93)	12.98 (*p* < 0.001) (biasη2 = 0.33)	6.99 (*p* = 0.002) (biasη2 = 0.21)
	Waitlist	60.95 (2.96)	59.91 (5.56)	60.56 (3.89)		
SAS^h^	CBT-P	62.69 (5.86)	57.22 (6.36)	58.24 (6.89)	20.20 (p < 0.001) (biasη2 = 0.43)	27.61 (*p* < 0.001) (biasη2 = 0.51)
	Waitlist	62.03 (5.84)	62.41 (7.00)	62.07 (5.60)		

a CM, Concern over Mistakes subscale of Frost Multidimensional Perfectionism Scale.

b PS, Personal Standards subscale of Frost Multidimensional Perfectionism Scale.

c PE, Parental Expectation subscale of Frost Multidimensional Perfectionism Scale.

d PC, Parental Criticism subscale of Frost Multidimensional Perfectionism Scale.

e DA, Doubts about Actions subscale of Frost Multidimensional Perfectionism Scale.

f MFMPS, maladaptive aspects of Frost Multidimensional Perfectionism Scale, consist of four subscales (CM, DA, PC, PE).

g SDS, Self-rating Depression Scale.

h SAS, Self-rating Anxiety Scale.

i CBT-P, CBT with positive psychotherapy.

As seen in Table 2, results of repeated measure ANOVA design with 3 times (baseline, post, and follow-up) × 2 groups (CBT-P, WL) were presented. There were significant main effects of time and time×group interaction effects for CM, DA, MFMPS, SDS, and SAS. In addition, effect sizes for the interactions were large for these measures.

Independent sample comparisons (*T*-test) were used to evaluate differences between the two groups at T1, T2, and T3. The analysis of simple effects seen in Table 3 indicated that the groups displayed a significant difference between CBT-P and WL in post-intervention for CM, DA, MFMPS, SDS, and SAS (*p* < 0.05). In reducing perfectionism (CM, and DA subscales of the FMPS), depression (SDS), and anxiety (SAS), the CBT-P was significantly more effective than WL.

**Table 3:** Between-group effect sizes at baseline (T1), post-intervention (T2), and 8-week follow-up (T3).

	Baseline			Post-intervention			Follow-up		
	*t*	*p*	Cohen’s *d*	*t*	*p*	Cohen’s *d*	*t*	*p*	Cohen’s *d*
*CM*									
CBT-P vs. Waitlist	0.37	0.713	0.10	−3.61	< 0.001	−0.96	−2.34	0.023	−0.63
*PS*									
CBT-P vs. Waitlist	0.40	0.689	0.11	0.65	0.519	0.17	0.07	0.948	0.02
*PE*									
CBT-P vs. Waitlist	−0.68	0.502	−0.18	−0.88	0.381	−0.24	−1.17	0.247	−0.31
*PC*									
CBT-P vs. Waitlist	0.40	0.693	0.11	−0.66	0.515	−0.18	−0.41	0.684	−0.11
*DA*									
CBT-P vs. Waitlist	0.63	0.50	0.17	−2.83	0.007	−0.76	−2.19	0.033	−0.59
*MFMPS*									
CBT-P vs. Waitlist	0.24	0.812	0.06	−3.35	0.001	−0.90	−2.50	0.015	−0.67
*SDS*									
CBT-P vs. Waitlist	−0.72	0.477	−0.19	−2.77	0.008	−0.74	−2.99	0.004	−0.80
*SAS*									
CBT-P vs. Waitlist	0.42	0.675	0.11	−2.90	0.005	−0.78	−2.29	0.026	−0.61

To examine whether treatment efficacy was maintained at the 8-week follow-up, a paired t-test was conducted for all measures. To identify treatment effectiveness, a paired t-test was conducted first between baseline and post-intervention in the two groups (CBT-P, WL) and then between post-intervention and follow-up, and finally between baseline and follow-up (Table 4). These results show that the intervention group displayed a significant reduction in depression and anxiety symptoms, and perfectionism at post-intervention. In CBT-P, the effect sizes (Cohen’s d) were large for CM, MFMPS, SDS, and SAS, and were moderate for DA. The effect was maintained at the 8-week follow-up compared with the effect at baseline, but the long-term effect was not satisfactory compared with the post-intervention effect.

**Table 4:** Within-Groups effect size at baseline (T1), post-intervention (T2) and 8-week follow-up (T3) in the intervention group and waitlist group.

	Baseline to post-intervention			Post-intervention to follow-up			Baseline to follow-up		
	*t*	*p*	Cohen’s *d*	*t*	*p*	Cohen’s *d*	*t*	*p*	Cohen’s *d*
*CM*									
CBT-P	5.73	<0.001	1.10	−2.74	0.011	−0.53	5.63	<0.001	1.08
Waitlist	−1.05	0.303	−0.20	1.16	0.255	0.22	−0.50	0.621	−0.09
*PS*									
CBT-P	1.03	0.311	0.20	0.95	0.352	0.18	2.01	0.055	0.39
Waitlist	1.30	0.204	0.24	−1.28	0.210	−0.24	0.58	0.565	0.11
*PE*									
CBT-P	1.72	0.097	0.33	−0.21	0.839	−0.04	2.02	0.054	0.39
Waitlist	0.65	0.520	0.12	−1.41	0.169	−0.26	−0.25	0.805	−0.05
*PC*									
CBT-P	1.73	0.095	0.33	−1.04	0.306	−0.20	1.07	0.294	0.21
Waitlist	−0.99	0.330	−0.18	0.00	1.000	0.00	−1.46	0.155	−0.27
*DA*									
CBT-P	3.97	<0.001	0.76	−2.73	0.011	−0.53	3.45	0.002	0.66
Waitlist	−0.48	0.638	−0.09	−0.15	0.882	−0.03	−0.74	0.467	−0.14
*MFMPS*									
CBT-P	6.83	<0.001	1.32	−3.55	0.001	−0.68	6.78	<0.001	1.30
Waitlist	−1.27	0.216	−0.24	0.33	0.741	0.06	−1.68	0.103	−0.31
*SDS*									
CBT-P	6.02	<0.001	1.16	−2.98	0.006	−0.57	5.03	<0.001	097
Waitlist	1.26	0.217	0.24	−1.12	0.272	−0.21	0.85	0.402	0.16
*SAS*									
CBT-P	7.63	<0.001	1.47	−3.18	0.004	−0.61	5.68	<0.001	1.09
Waitlist	−1.04	0.307	−0.19	0.79	0.438	0.15	0.10	0.924	−0.02

## Discussion

Group cognitive behavior therapy with positive psychotherapy intervention was first used to reduce the symptoms of depression or anxiety by targeting maladaptive perfectionism in Chinese students. The results supported the practicability and effects of this group intervention. Through the eight-week group intervention, the main effect of time and the time × group interaction effect were significant for the MFMPS, SDS, and SAS scores.

In addition, the observed effect sizes were comparable to those obtained from CBT treatment trials for perfectionism (Handley et al., 2015; Wade et al., 2019). For example, a previous waitlist-controlled trial for individual ACT reported between-group post-intervention Hedges’ gs ranging from 0.42 to 1.05 for FMPS scores (Ong et al., 2019); corresponding effect sizes in the present study ranged from 0.74 to 0.96. This finding supports the conclusion of a meta-analysis that psychological interventions can reduce perfectionism and anxiety and depression symptoms, and the theory that perfectionism interventions can effectively reduce a series of psychiatric disorder symptoms (Lloyd et al., 2015).

The introspection regarding the negative influence of high standards, parents’ expectations, and the origin of perfectionism, and the training on how to deal with failure and change procrastination habits were designed in the intervention. There were significant group differences for CM and DA at post-intervention and follow-up but there were no significant group differences for PS, PE, and PC at post-intervention and follow-up. This verified that individuals might show resistance to changing their personal standards, which they view as a dimension of their personality, consistent with the outcomes of previous treatment (Ong et al., 2019). Individuals in the intervention group could still set higher standards but limit them to some areas rather than all areas. In addition, high standards may be adaptive and were associated with positive outcomes in some studies (Bieling et al., 2004; Stoeber and Otto, 2006; Gäde et al., 2017). However, they were not included in maladaptive perfectionism in this study, so individuals may not need to change PS to live a meaningful life. Because perfectionists themselves were mainly involved in the intervention, the source of perfectionism was traced, and participants understood why their parents had high expectations for them, but the parents did not take part in the intervention. We hypothesize that this was why there was no significant change in the scores of PE and PC.

In this study, by targeting transdiagnostic maladaptive perfectionism, the risk and maintenance factors in the treatment of depression and anxiety disorders can be reduced. Compared with previous intervention studies in China, critical scores from the MFMPS, SDS, and SAS were used to select participants, and treatment based on a CBT protocol for depression and anxiety was conducted in the intervention. The subdimensions of perfectionism with a high negative correlation to psychiatric symptoms were targeted. Participants changed for the following reasons at post-intervention and follow-up. They had the opportunity to view unreasonable demands or expectations for each other. The negative influence of perfectionism was clearly defined with a focus on difficulty making a decision, procrastination habits, feelings of frustration, feelings of insecurity, low self-evaluation, fear of failure, etc. By intervening in these aspects, automatic thinking caused negative reactions to be discovered and perceived. Behavior experiments were used to challenge rigid perfect beliefs acting as trustful faith. Intermediate beliefs with maladaptive perfectionism were identified and changed. An alternative adaptive belief was delivered to participants through cognitive restructuring: reacting according to the actual present situation rather than a kind of rigid pattern. Training for procrastination because of avoidance behavior and fear of failure was provided to explore the influence of family members and to increase self-acceptance. Then, their own advantages and strengths could be used to recognize themselves, enhance their confidence and determination, and cultivate positive thinking habits.

When the effect of the intervention was evaluated, we not only compared whether the difference between the intervention group and the waitlist group was significant at baseline, post-intervention, and follow-up but also added effect size indicators, excluding the influence of sample size in this study. Group CBT is an appealing psychological intervention given its potential cost- and time-effectiveness for treating many patients and is viewed as less cost-effective and as more cost-effective than individual CBT (Okumura and Ichikura, 2014).

The deficiencies of this study were as follows. The sample size was just approaching the minimal demand. The change in perfectionism was a gradual process and some participants would have graduated after 8 weeks of intervention, follow-up in this study was at 8 weeks. The effect sizes were maintained with a declining trend and it is necessary to design more long-term and better intervention protocols for maladaptive perfectionism. The participants were selected by the critical scores of the MFMPS, SDS, and SAS scales but were not diagnosed by DSM-V. Those who volunteered to participate might have higher motivation to change. As stated before, perfect thinking and behavior patterns are born in the personality dimensions of maladapted perfectionists, and they are long-term and habitual. Perfectionists may show resistance to change, and the relevant psychiatric symptoms easily relapse. A short-term intervention of 8 weeks may produce a preliminary change in a nonclinical sample, and the validity of the results will not generalize to clinical samples. The intervention will focus on high perfectionist tendencies and clinical samples with diagnoses in the future. If perfectionism is a maintaining mechanism of AXIS Ι Disorders in DSM-V, then it is more effective to aim at perfectionism, but more research is needed in the future. Finally, despite the intervention being manualized and supervised, treatment adherence measures were not used; thus, it is not known the extent to which therapists adhered to the treatment protocol.

The strengths of the study are that it is the first RCT with adequate power to check the effectiveness and prognosis of group CBT with positive psychotherapy in targeting core underlying factors such as perfectionism rather than disorder-specific symptoms. The results contribute further evidence of a transdiagnostic effect of group CBT with positive psychotherapy for perfectionism, with significant reductions in depression and anxiety (Shafran et al., 2017).

## Data availability statement

The raw data supporting the conclusions of this article will be made available by the authors, without undue reservation.

## Ethics statement

Ethical review and approval was not required for the study on human participants in accordance with the local legislation and institutional requirements. The patients/participants provided their written informed consent to participate in this study.

## Author contributions

ZZ conducted the data collection, data analysis, and data curation and wrote the original draft. XZ wrote, reviewed and edited the manuscript. All authors drafted the methodology and conceptualized the aims. All authors approved the submitted version.

## Conflict of interest

The authors declare that the research was conducted in the absence of any commercial or financial relationships that could be construed as a potential conflict of interest.

## Publisher’s note

All claims expressed in this article are solely those of the authors and do not necessarily represent those of their affiliated organizations, or those of the publisher, the editors and the reviewers. Any product that may be evaluated in this article, or claim that may be made by its manufacturer, is not guaranteed or endorsed by the publisher.
